# Exploring students' perceptions on the use of significant event analysis, as part of a portfolio assessment process in general practice, as a tool for learning how to use reflection in learning

**DOI:** 10.1186/1472-6920-7-5

**Published:** 2007-03-30

**Authors:** Andrew J Grant, Jan D Vermunt, Paul Kinnersley, Helen Houston

**Affiliations:** 1 Department of General Practice, Cardiff University, Centre for Health Sciences Research, 3^rd ^floor, Neuadd Meirionedd, Heath Park, Cardiff, CF14 4XN, UK; 2 IVLOS Institute of Education, Utrecht University, P.O. Box 80.127, 3508 TC Utrecht, The Netherlands

## Abstract

**Background:**

Portfolio learning enables students to collect evidence of their learning. Component tasks making up a portfolio can be devised that relate directly to intended learning outcomes. Reflective tasks can stimulate students to recognise their own learning needs.

Assessment of portfolios using a rating scale relating to intended learning outcomes offers high content validity.

This study evaluated a reflective portfolio used during a final-year attachment in general practice (family medicine). Students were asked to evaluate the portfolio (which used significant event analysis as a basis for reflection) as a learning tool. The validity and reliability of the portfolio as an assessment tool were also measured.

**Methods:**

81 final-year medical students completed reflective significant event analyses as part of a portfolio created during a three-week attachment (clerkship) in general practice (family medicine). As well as two reflective significant event analyses each portfolio contained an audit and a health needs assessment.

Portfolios were marked three times; by the student's GP teacher, the course organiser and by another teacher in the university department of general practice. Inter-rater reliability between pairs of markers was calculated. A questionnaire enabled the students' experience of portfolio learning to be determined.

**Results:**

Benefits to learning from reflective learning were limited. Students said that they thought more about the patients they wrote up in significant event analyses but information as to the nature and effect of this was not forthcoming.

Moderate inter-rater reliability (Spearman's Rho .65) was found between pairs of departmental raters dealing with larger numbers (20 – 60) of portfolios. Inter-rater reliability of marking involving GP tutors who only marked 1 – 3 portfolios was very low.

Students rated highly their mentoring relationship with their GP teacher but found the portfolio tasks time-consuming.

**Conclusion:**

The inter-rater reliability observed in this study should be viewed alongside the high validity afforded by the authenticity of the learning tasks (compared with a sample of a student's learning taken by an exam question). Validity is enhanced by the rating scale which directly connects the grade given with intended learning outcomes.

The moderate inter-rater reliability may be increased if a portfolio is completed over a longer period of time and contains more component pieces of work.

The questionnaire used in this study only accessed limited information about the effect of reflection on students' learning. Qualitative methods of evaluation would determine the students experience in greater depth. It would be useful to evaluate the effects of reflective learning after students have had more time to get used to this unfamiliar method of learning and to overcome any problems in understanding the task.

## Background

### Portfolios

Portfolios enable students to gather evidence of their learning via a series of tasks. The component tasks can be designed to meet intended learning outcomes. The work collected in the portfolio provides material for the student to review their learning and can be used as a basis for assessment. Studies with pre-service teaching students found that keeping a portfolio enabled students to connect theory with practice and increased their awareness of their strengths and weaknesses as future teachers [1].

When introduced to portfolio learning most students find it unfamiliar and difficult and benefit greatly from some instruction in the theory and method of portfolio learning [2,3]. Getting over the initial difficulties of keeping a portfolio is cited as part of the satisfaction that comes from persevering with this learning strategy [4].

### Mentoring

Although the product of portfolio learning is extremely valuable, the process of developing it is a vital part of students' learning. Learners will benefit from regular input from a mentor while engaging in portfolio learning [2,5,6]. A mentor can help to support the student as they get established in the process of portfolio learning. They can also oversee students' learning, give feedback, and help them to identify learning needs, challenging them when they avoid difficult areas. To be lasting and pervasive a method of learning must involve affect as well as intellect [7] and a mentor can help students deal with the emotional content of learning. Students can act as mentor for each other in peer groups where they can often learn from fellow students' portfolio entries as well as from their own.

### Reflection

John Dewey first described reflection in relation to learning in his book "How We Think" [8]. He said it differed from other sorts of thinking because it was initiated by a sense of unease in the learner when they realised that their knowledge was incomplete or inaccurate and that it differed from other forms of thought in having a definite outcome. Reflection can enable students to take their learning to a deeper level [9].

There is little in the literature on the use of reflection in undergraduate medical education [5] but many studies exist describing its use in initial teacher education and in the education of nurses [9-11].

### Significant Event Analysis

One of the challenges when introducing students to reflective learning strategies is to provide enough structure that they don't feel lost without making the process mechanistic. Significant event analysis forms a template for students' reflection. Based on the Critical Incident Technique [12] it provides a structure for learners who are unfamiliar with reflective learning. The learner sets out his/her reflection under four sequential headings: what happened?, Reflection, Identified learning needs and Learning plan. A qualitative study of medical students' significant event analyses, explored conflicts and coping strategies of students completing significant event analyses within portfolios kept during a fourth year general practice attachment [2] The authors found that many students were unwilling to discuss their feelings and were reluctant to criticise their teachers when their work was going to be marked. Two factors deemed of greatest importance by the students were the time involved in completing the significant event analyses and the strength of the mentoring relationship with the GP teacher.

### Audit

Clinical Audit enables reflection on performance [13]. Clinical audit will be a requirement for all the students in this study during their lives in clinical practice. In this study students carried out an audit of communication between primary and secondary care. This topic was chosen because they would all soon be working as junior hospital doctors when the quality of the information they sent out to general practitioners would have a direct bearing on the care their patients received.

### Heath Needs Analysis (HNA)

One of the aims of this attachment was that students should develop an understanding of the health needs of the population in the local area. A health needs analysis was included as one of the portfolio tasks to enable them to achieve this goal.

### Using portfolios in Assessment

Portfolios potentially enable authentic assessment of the students' work. The effect of portfolio assessment on students' learning depends on how well the portfolio tasks are designed but if carrying them out involves the students engaging in learning as defined in the learning objectives content validity will be high.

By their very nature portfolios are highly individual making assessment of them difficult. Only when markers have a shared understanding of the objectives of the curriculum and apply them consistently will there be an acceptable level of construct validity. A rating scale written with reference to the learning outcomes can help examiners mark portfolios consistently [14] [see additional file 1].

Attempts to increase the inter-rater reliability by standardising portfolios or to make marking more objective endanger the individuality of their content [5]. Increasing the number of markers (raters) will increase reliability but there are obvious manpower implications

Opinions vary whether or not portfolio assessment can be valid and reliable in different settings [15,16]. Work on portfolio assessment of schoolchildren [17] and preservice teachers [18] has led authors to conclude that portfolio assessment can be valid and reliable but this contrasts with the opinion of others working with general practice trainers [15]. Marking grids or profiles have been developed to define to the marker what would be acceptable as evidence that a particular learning outcome has been achieved to a satisfactory or excellent standard [14,19].

### Research questions

This study explored the use of significant event analyses as a reflective learning tool as part of a learning portfolio created by final-year medical students on a three-week attachment in general practice (family medicine). It also set out to discover whether portfolios could form the basis for valid, reliable assessment in this context.

## Methods

### Subjects

Subjects were 81 medical students in the final year at Imperial College Medical School, London. The participants in this study had kept a portfolio including significant event analyses during one previous attachment in general practice [2].

### The attachment

Students involved in this study were in their final year at Imperial College School of Medicine in London. They had already completed one attachment in general practice in their fourth year (of a five-year curriculum) where they had completed a portfolio which included significant event analyses [2].

The portfolio was created on a three-week attachment (Clerkship) in General practice (Family Medicine). The first day-and-a-half were spent at the medical school where students were introduced to the attachment and carried out some agenda setting exercises. On the last day they returned to take part in a debrief when they had to submit a portfolio containing an audit, a health needs analysis and two significant event analyses

The students spent the remainder of the three-week period in general practices all over the United Kingdom. There was never more than one student per practice and students were resident with the GPs and their families. Students were encouraged to make the most of the opportunity to see many patients and to practice their clinical skills. As well as completing the portfolio tasks students sat in with the doctors in the practice where they were able to examine patients under supervision and receive one-to-one feedback.

Student surgeries (clinics) were an important part of the attachment. Students saw patients first in a room on their own where they took a relevant history and carried out any appropriate examination. When the student was happy that they had progressed as far as they could on their own they called their GP teacher who asked for their diagnosis and plan of action. Where appropriate students examined or re-examined the patient in front of the GP teacher enabling feedback to be given. Students did not carry out any intimate examination nor did they initiate any treatment or investigation until their GP teacher had seen the patient.

### Agenda setting

Students were asked to prepare 2 items to enable them and their GP teachers to plan the attachment around their individual needs; a mini curriculum vitae (résumé) and a list of their learning objectives for the attachment.

### Significant event analysis (SEA)

The completed portfolio had to contain reflective write-ups (significant event analyses) of at least two clinical encounters. The students were encouraged to write up encounters they felt were significant to their learning and did not confine themselves to events where they had exposed a gap in their knowledge or where something had gone wrong. The significant event analysis headings encouraged students to examine their knowledge and identify learning needs through reflection.

### Health needs analysis

Students completed a health needs analysis of the area. They were encouraged to collect data from as wide a variety of sources as possible, to write a brief report and to include one recommendation how the practice may address a local health need. In carrying out the task students met a wider spectrum of people in primary care and public health than they might otherwise have done.

### Audit

Because of the limited time available students were not required to include more than ten patients in their clinical audit.

### Course Guide

To complement the information given during the introduction the students were given a course guide that gave them instructions how to complete all of the portfolio tasks. Some useful references were included.

### Marking

We used a three-point scale for marking the portfolio components; better than expected (Be), Expected (Ex.), and Refer (Re) this was similar to the 3 grades "Possible Distinction, Achieved and Not Achieved" developed by Usherwood and Hannay [14]. If a student was given the refer (Re) grade they would have to carry out some remedial work and could not get their degree (licensing qualification) until this had been successfully completed.

### Rating scales

For each of the three tasks rating scales provided raters with benchmarks for each grade [see additional file 1]. The rating scales give a definition of performance at each of the portfolio tasks at Be, Ex and Re levels.

Each portfolio was marked three times; by the student's GP teacher (GPT), one of three course tutors, and the Course organizer.

The rating scale and its underlying constructs were discussed among the course tutors. Written instructions in the use of the rating scale were given to the GP teachers.

### Inter-rater reliability

For the purpose of calculating inter-rater reliability three pairs of raters were formed; GP teacher × Course tutor, Course tutor × Course organizer, & GP teacher × Course organizer (see table 1). Spearman's Rho was used to compute inter-rater reliability for each pair.

**Table 1:** Rater groups

***Rater***	***Number of markers in group***	***Number of portfolios marked by each marker***	***Involved in developing course and rating scale***
GP Teacher	>50	1–3	-
Course tutor	3	20	++
Course organizer	1	60	++

Number of raters in each group and degree of involvement with course development

#### The Questionnaire

In order to explore the effect of the portfolio on students' learning we developed a questionnaire (see table 2) where students were asked to respond to 18 statements on a five-point Likert scale (1 = strongly disagree to 5 = - strongly agree). Questions were grouped under six headings. Five of these headings (agenda setting, SEA process, audit, health needs assessment and mentoring) covered student learning in relation to the processes involved in constructing the portfolio. A sixth (metacognition) explored students awareness of changes in their learning as a result of completing the significant event analyses. Students were also asked to respond to three statements in free text; "what I liked best about the portfolio was", "what I liked least about the portfolio was" and "the portfolio would benefit from being changed in the following ways". In order to maximise content validity the questionnaire was developed in consultation with the course tutors and was piloted with a group of students not otherwise involved in the study.

**Table 2:** Rating questionnaire results

*Question*	*Mean*	*SD*
**Agenda setting **(alpha = .60)		
1. Writing down my past clinical education helped me identify my learning needs for this attachment	2.12	1.06
2. I feel I learned more because I wrote down my learning objectives on the first day	2.5	1.35
19. Having my past clinical education written down helped my GP teacher to cover the topics I most needed to learn	2.13	1.19
		
**SEA Process **(alpha = .70)		
3. SEAs provide a useful framework for learning	3.0	1.19
7. Completing the SEAs stimulated me to look things up	2.45	1.17
13. I had to think more about the consultations I wrote up for my SEAs	3.70	1.09
		
**Metacognition **(alpha = .79)		
6. By completing the SEAs I have increased the amount I have learned.	2.58	1.24
11. The SEAs enabled me to identify what I still need to learn	2.54	1.27
16. The SEAs enabled me to identify what I already know	2.93	1.24
		
**Audit **(alpha = .61)		
10. The audit was enjoyable	2.78	1.20
15. The audit was interesting	3.23	1.20
18. I now know more about communication between primary and secondary care.	3.86	.97
		
**Health Needs Assessment **(alpha = .77)		
5. Completing the HNA has given me a clearer idea of the health needs of the population	3.46	1.25
12. The HNA was interesting	3.18	1.61
14. The HNA was enjoyable	2.81	1.25
		
**Mentoring **(alpha = .52)		
4. I learned more because I discussed my learning objectives with my GP teacher	3.07	1.33
8. My GP teacher helped a lot in my learning throughout the attachment	4.46	.69
9. Discussing the SEAs with my GP teacher increased the amount that I learned	3.09	1.25

(n = 78)

Scale; 1 = strongly disagree, 2 = disagree, 3 = no feelings either way, 4 = agree, 5 = strongly agree. Percentage of ratings for each point on Likert scale as well as mean and standard deviation.

Internal consistency (Cronbach's alpha) shown for each sub-group

We analysed the internal consistency of the questionnaire as a whole and for each of the six headings by computing Cronbach's alpha. The mean and standard deviation for the responses to each question were calculated. We analysed the written responses to the three questions under the same six headings as the questionnaire items; Agenda setting, Significant Event Analysis, Metacognition, Mentoring, Health Needs Assessment, and Audit.

#### Ethical approval and consent

This study involved evaluation of an innovation into the undergraduate medical curriculum. As it did not involve patients or employees of the National Health Service it was not subject to ethical approval by the research ethics committee network. At the time that the study was carried out no mechanism existed at Imperial College for ethical approval of educational research involving medical students.

The questionnaire was distributed on the last day of the attachment, completion was voluntary.

## Results

We distributed 81 questionnaires of which 78 were returned (96% response rate). Sixty-two of the students' portfolios were triple marked

### Inter-rater reliability

Inter-rater reliability (see table 3) showed wide variation. The course tutor × course organizer pair had the highest correlation of 0.65. The course tutor × GPT and the GPT × course organizer pairs had Rho values of 0.32 and 0.16 respectively.

**Table 3:** Inter-rater reliabilities.

Rater pair	Total (3 tasks)
GPT × Course tutor	.32
GPT × Course organizer	.16
Course tutor × Course organizer	.65

Spearman's correlation coefficient Rho

### The questionnaire

Internal consistency (Cronbach's alpha) for all 18 items was 0.82. For internal consistency for the individual headings see table 2.

### Mentoring

The item scored highest was "My GP teacher helped me a lot in my learning throughout the attachment" (4.46)

### SEA process

Students had to think more about the patients they wrote up for the significant event analyses but the other items under this heading did not elucidate how this was facilitated.

### Agenda setting

Agenda setting items were not rated highly despite a number of students saying, in free text that they helped them recognise their strengths and weaknesses and helped them plan for the attachment.

### Health needs assessment and audit

Completing the HNA gave students a clearer idea of the health needs of the population and the topic of the audit (primary/secondary care communication) increased students' knowledge in this area.

### Free text comments

Students were able to broaden their feedback by responding to the last three questions which required responses in free text. Comments about the portfolio overall included;

"*Allowed me to voice my opinion and write creatively*"

"*The written work would benefit if it were less intense – I enjoyed the self-reflection probably because I had something to write about*."

"*Could not be improved upon*."

### Significant event analyses

Students held strong opinions for and against the significant event analyses. Seven students identified significant event analyses as the portfolio component they liked best while ten liked them least. Nine students wanted fewer significant event analyses (there were only two). One student commented that the structure given for the SEAs was too prescriptive.

Comments about the significant event analyses included;

"*Like to choose my own topics for the SEAs because they make me think*"

"*Good learning tool, could have done 10 – 15*"

"*A bit patronising, like being back at school*,"

Many of the suggested changes related to the way the template for the significant event analysis had been set out in the course guide, leaving too little space.

### Time

A major issue for students was the time taken to complete the portfolio. Nine students wrote that the volume of written work was too much for the time available and that it took them away from time with patients.

### Agenda setting

Despite the negative response to the agenda setting exercises three students listed them as the thing they liked most about the portfolio, compared to seven who liked them least. Two wrote that the learning objectives were a waste of time one of those because they had not been discussed with their GP teacher.

### Audit and Health Needs Analysis

Five students liked the health needs analysis best while two liked it least. Individual comments indicated that it had given insight into the practice area and had been relevant and interesting.

Eleven students liked the audit best. One student said that they had learned a lot by carrying it out but another complained that it was dull because everyone had to do the same topic

### Fabrication

Of particular note is that one student made up the cases on which they based their significant event analyses and another said that they had written what they thought the GP teachers wanted to read.

## Discussion

### Study findings

On this three-week attachment the students did not report benefits to their learning from using a portfolio with reflective significant event analyses beyond thinking more about patients they wrote up in their significant event analyses. Inter-rater reliability was moderate at 0.65 for pairs of raters marking 20 or more portfolios but was lower for pairs involving GP teachers who marked only small numbers (two or three).

The next two sub sections, *Effects of the portfolio on learning*, and *Validity and Reliability *set out the study findings in relation to the two research questions; the effect of the reflective portfolio on learning and the validity and reliability of the portfolio as an assessment tool.

### Effects of the portfolio on learning

Our findings are consistent with previous published work on reflective learning in general practice. Students rated their mentoring relationship with their GP teacher most highly and found the process time-consuming [2]. Writing the Significant Event Analyses stimulated students to think more about the patients they wrote up but did not stimulate them to look things up or raise their awareness of their own knowledge.

Despite having rated the help they received from their GP teacher highest no further information about the mentoring role was accessed. There were a number of places in the process where problems may have occurred. Very little time was available for instructing students about reflective learning during the course introduction. The time needed for learners to get used to reflective learning before they experience its benefits has been described in the literature and despite the students having used reflective learning before they did not find it beneficial. Although written material had been sent to all the GP tutors about the mentoring role many may not have had much personal experience of reflective learning.

### Validity and Reliability

The content validity of this portfolio assessment is supported by the use of a rating scale taken directly from the intended learning outcomes which is supported by published work [14]. The study also measured validity indirectly by asking students what effect portfolio assessment had on their learning. Theoretical validity can, however, be undermined in practice. The students who made up their significant event analyses and wrote what they thought the teacher wanted to read undermined validity. Having to hand in the significant event analyses for assessment had driven the students to do what they thought would gain them highest marks -thereby short circuiting the intended learning process.

While .65 is not as high as the reliability found in other forms of assessment this has to be viewed in combination with the validity of portfolio assessment and its direct connection to intended learning outcomes. The level of inter-rater reliability found in this study compares favourably with .51 found by Usherwood and Hannay [14] for their criterion-referenced profile (rating scale). It is not surprising that the highest level of inter-rater reliability was found in a pair of raters both of whom had marked the largest number of portfolios.

Despite the mentoring they provided being highly rated by students the very low inter-rater reliability of their marking suggests that the GP teachers did not share a clear understanding of the aims of the portfolio as a support for learning and an assessment tool with the other raters.

### Limitations of this study

The short time period allocated for this study limited students' opportunity to adapt to this relatively unfamiliar learning strategy. It appears that many students did not experience the described benefits of portfolio or reflective learning described in the literature [18]. This may be due to their not having had sufficient time to adapt to using reflection or to experience its benefits for themselves. Had we been able to evaluate this group's experience over a longer period with regular feedback from the mentors they may have overcome their initial difficulties and experienced the benefits of reflective learning firsthand.

The difference in preparation of raters in the use of the rating scale may, to some degree, explain the observed differences in inter-rater reliability and must be recognised as a possible source of bias. The course organiser and course tutors had been able to discuss the rating scale and its underlying constructs whereas the GP tutors had only been sent the scale by post with written guidance on its use.

### Implications for future practice

It is necessary to minimise the effect of assessment of the portfolio on students' learning. A portfolio created over a longer period of time would allow the students to include a greater number of pieces of work as evidence of satisfactory progress thereby reducing the need to perform well on any one assignment. Students involved in this study were still relatively unfamiliar with reflection in learning. It is likely that, in time, they would develop confidence in their ability to carry out reflective pieces of work satisfactorily without resorting to fabricating patients or writing to please the assessor.

Clearer information to GP tutors on the aims of the portfolio and on the use of the rating scale may have increased inter-rater reliability in this group.

### Further research

A longer study would show whether more students would experience the theoretical benefits of portfolio learning with more time and supportive mentoring. A longer study would also make it possible to discover whether a portfolio with more component parts would result in higher inter-rater reliability.

Using a questionnaire in this study restricted the data obtained to responses to the questions deemed most important by the authors. The questions requiring free text responses did provide some opportunity for students to express their own ideas but it did not enable us to probe further the responses we received. A qualitative approach to evaluating the effects of portfolio learning may obtain information in greater depth about the experience of portfolio learning at the level of the individual student. Research using the nominal group technique may access the opinion of a large number of students while minimising the peer group effect but a study involving one-to-one interviews would obtain the most in-depth data.

## Conclusion

Students did not report benefits from portfolio learning on this brief attachment beyond having had to think more about patients they wrote up.

The short timeframe of this study may not have allowed students an opportunity to overcome initial difficulties and to experience the benefits of reflective learning firsthand.

Portfolio assessment achieved moderate levels of inter-rater reliability when marked by teachers who marked large numbers (< 20) of portfolios. A rating scale derived from intended learning outcomes supported high content validity in portfolio assessment.

## Competing interests

The author(s) declare that they have no competing interests.

## Authors' contributions

AG collected and analyzed the data and wrote and revised the manuscript. JV supervised the design of the study, data collection and analysis and revised the manuscript. PK read and revised the manuscript. HH read and revised the manuscript. All authors read and approved the final manuscript.

## Pre-publication history

The pre-publication history for this paper can be accessed here:



## Supplementary Material

Additional File 1 MS Grant et al portfolio assessment final 230307 additional file.doc. Rating scales for Significant event analysis, health needs assessment and audit. Click here for file
