# Supplementary material for: Exploring students' perceptions on the use of significant event analysis, as part of a portfolio assessment process in general practice, as a tool for learning how to use reflection in learning
Source: BMC Med Educ. 2007 Mar 30;7:5. doi: 10.1186/1472-6920-7-5 (PMC1852102; doi:10.1186/1472-6920-7-5)
Supplement: Additional File 1 — MS Grant et al portfolio assessment final 230307 additional file.doc. Rating scales for Significant event analysis, health needs assessment and audit. [file 1472-6920-7-5-S1.doc]

**Exploring students’ perceptions on the use of significant event analysis, as part of a portfolio assessment process in general practice, as a tool for learning how to use reflection in learning**

Andrew Grant^§^, Jan D. Vermunt, Paul Kinnersley & Helen Houston

**Additional file 1 – Rating scales for Significant event analysis, health needs assessment and audit**

Heath Needs Assessment rating scale for

| Better than Expected (Be)   - Numerous interviews completed (including some from the desirable/optional list) . - Student demonstrates a thorough understanding of information sources. - Health problems identified and well supported by local information. - Full discussion of how information was weighed. - Student has reflected in some depth on the extent the practice structure meets the needs of the locality. - Initiatives and suggestions are sensible and well focused. - Report shows an in depth understanding of the health problems of the locality. | Expected (Ex)   - Essential interviews completed. - Student demonstrates an awareness of information sources available to practices. - Health problems identified and supported by local information. - Some discussion of how information was weighted. - Student discusses the extent to which the practice structure reflects the needs of the locality. - Initiatives and suggestions for change offered. | Refer (Re)   - Essential interviews not completed. - Student has not established sources of information available to practices. - Report minimal or not done. - Health problems not identified and/or not substantiated by any local information. - Little or no discussion on how information was weighted. - Little or no reflection on the extent the practice structure meets the needs of the locality. - Initiatives and suggestions for change not made. |
| --- | --- | --- |

Significant Event analysis Rating scale

| Better than Expected (Be)   - Demonstrates a high degree of reflection - SEAs show a genuine understanding of general practice - Student shows an understanding of own learning needs - Descriptions are concise but clear and demonstrate excellent powers of observation - Reflection on experiences and feelings show honesty and self awareness - The evaluation and analysis are balanced, searching and constructively critical/self critical - The student is willing to explore his/her own values and attitudes - The learning plans are realistic and consistent with the analysis of the events. - The student has discussed the SEAs with their GP teachers and always includes of this and any new insights gained. - The SEAs are well presented and handed in on time | **Expected (Ex)**   - The significant events selected demonstrate an adequate grasp of general practice - Students some insight into their own learning need - Some descriptions lack clarity, evidence or observation. - Reflection is included but tends to lack details of thoughts and feelings provoked by the events. - Attempts at evaluation and analysis are present but may show limited understanding of some events, tending to be excessively critical/self critical. - There is little exploration of values and attitudes - The learning plans are vague, inappropriate or absent - Some events have not been discussed with the GP teacher with no explanation provided. - Presentation is adequate with some errors | **Refer (Re)**   - SEAs not completed - Shows a lack of commitment to independent learning. - The significant events are irrelevant or absent. Unable to demonstrate an understanding of general practice or self. - Descriptions are superficial or absent - Reflection is not demonstrated - Evaluation and analysis absent or consistently superficial or misplaces - Unwillingness to explore values and attitudes. - No learning plans - Presentation poor with frequent mistakes |
| --- | --- | --- |

Audit rating scale

| Better than Expected (Be)   - Reads like a recipe. - Write up could be used to explain the stages of the audit process to someone with no prior knowledge. Clear presentation of results. Thorough discussion of results/process. - Insight into any problems uncovered. Insightful recommendations for improvement. - Presentation of insights gained (a.) Into clinical audit. (b.) Into primary/secondary communication | **Expected (Ex)**   - Student demonstrates an understanding of the stages of audit. - Results presented. - Discussion of results – - explanation of implications and suggestions for improvement | **Refer (Re)**   - Audit has not been completed. - Understanding of the stages of audit not demonstrated. - Results either not presented at all or not in a way they can be clearly understood. - No discussion of the implications of the results or suggestions on how performance can be improved |
| --- | --- | --- |
